# Youth in the study of comparative physiology: insights from demography in the wild

**DOI:** 10.1007/s00360-020-01315-z

**Published:** 2020-10-22

**Authors:** Richard W. Hill, David A. Sleboda, Justin J. Millar

**Affiliations:** 1 Department of Integrative Biology, Michigan State University, East Lansing, MI 48824 USA; 2 Department of Physiology, McGill University, Montreal, QC H3G 0B1 Canada; 3 Big Data Institute, University of Oxford, Old Road Campus, Oxford, OX3 7LF UK

**Keywords:** Juveniles, Immatures, Young, Life tables, Survivorship, Mortality, Ontogeny, Development, Deevey

## Abstract

Of all the properties of individual animals of interest to comparative physiologists, age and stage of development are among the most consequential. In a natural population of any species, the survivorship curve is an important determinant of the relative abundances of ages and stages of development. Demography, thus, has significant implications for the study of comparative physiology. When Edward Deevey published his influential summary of survivorship in animal populations in the wild seven decades ago, he emphasized “serious deficiencies” because survivorship curves for natural populations at the time did not include data on the earliest life stages. Such data have accumulated over intervening years. We survey, for the first time, empirical knowledge of early-age survivorship in populations of most major animal groups in a state of nature. Despite wide variation, it is almost universally true that > 50% of newly born or hatched individuals die before the onset of sexual maturity, even in species commonly assumed to exhibit high early-age survivorship. These demographic facts are important considerations for studies in comparative and environmental physiology whether physiologists (i) aim to elucidate function throughout the life cycle, including both early stages and adults, or (ii) focus on adults (in which case early-age survivorship can potentially affect adult characteristics through selection or epigenesis). We establish that Deevey’s Type I curve (which applies to species with relatively limited early mortality) has few or no actual analogs in the real, natural world.

## Introduction

In the study of comparative physiology, an important dimension is comparison of the sequential stages of postnatal development within species. Already in the early nineteenth century, Edwards (1824) observed that young mammals and birds—compared with adults of their species—are less capable of maintaining a high body temperature in cold surroundings. In more recent decades, comparative studies of the stages of development have become more detailed, and they have addressed a wide diversity of animals and levels of organization. To illustrate with a few examples, Hill (1976) detailed the radical changes in metabolic rate and thermoregulatory competence that take place as newborn mice (*Peromyscus leucopus*) mature to adulthood—including the remarkable fact (Hill 2017) that early-age nestlings employ apneic pulmonary O_2_ uptake to survive periods when they lapse into profound hypothermia. Fuiman and Batty (1997) demonstrated that the swimming performance of herring (*Clupea harengus*) is strongly affected by temperature-dependent changes in water viscosity when the fish are 1 month old, although unaffected when they are adult. At a molecular level, Matoba et al. (2000) demonstrated that dramatic changes occur in gene expression within a group of > 1800 genes in the cerebellum of mice (*Mus domesticus*) between birth and 6 weeks of age, including large expression increases at and following the age of weaning for many genes coding neurotransmitter receptors, ion channels, and ion transporters. Also at a molecular level, Lestyk et al. (2009) documented major advances in muscle tissue maturation during the postnatal development of hooded seals (*Cystophora cristata*); these advances—which included large increases in myoglobin concentration and acid buffering capacity—helped enable the seals to dive far longer when adult than when young.

Traditionally, most studies in comparative physiology have been carried out on adults. Nonetheless, as the cases just noted illustrate, there has long been a relatively small, parallel, and multi-dimensional effort to understand the physiology of youth. However meager this effort might have been in some respects, it has established one central point beyond doubt: The young typically differ from the adults of their species in their physiological capabilities and often interact with environmental variables in fundamentally different ways than adults. In fact, of all the properties of animals pertinent to studies in comparative physiology, age and stage of development are frequently among the most consequential.

Thus, when comparative physiologists conduct studies aimed at understanding animals in their natural environments, demography is potentially of key significance. Consider the potential implications of demographic information, using as an example a species that undergoes four postnatal stages of development (stages 1–4) prior to adulthood. In a study of such a species in relation to its natural environment, physiologists might adopt either of two complementary perspectives, an “adult perspective” or an “all-individuals perspective.” In the adult perspective, the physiology of adults is the principal focus and only adults are studied. Demography is still potentially important, however, because each adult had to pass through stages 1–4 to reach adulthood, and demography provides quantitative insight into the rigors faced at each of these stages. For example, if demography reveals that the death rate is 90% during stage 1, physiologists studying adults will know that the properties of the adults may have been significantly affected by strong natural selection or epigenetic effects in stage 1. Demography is of far greater immediate significance when physiologists adopt an all-individuals perspective in which they seek to understand the physiology of all individuals born into a population—not only the adults but also the never-adults (individuals that die prior to adulthood). Demography reveals the numbers of individuals in each category. In the all-individuals perspective, the physiology of the young is of direct, immediate significance. Consider, for example, individuals that die in stage 1. For them, the only physiology that matters is the physiology of stage 1. That physiology *is* their physiology. The physiology of individuals that die in stage 4 is a composite of the pre-adult physiologies expressed in stages 1–4, in the sense that such individuals live through all four preadult stages before dying. Again, demography reveals the numbers of individuals in the successive stages.

With these considerations in mind, we have surveyed what is known today about the early-age demography of animals in the wild. This information is essential for environmental physiologists (physiological ecologists) and for all other comparative physiologists who focus on the on the relationships of animals to their natural environments. We stress that our exclusive interest in this paper is animals in natural populations in their natural environments: populations that we refer to as “natural populations,” “wild populations,” or “populations in a state of nature.” By these terms, we refer to populations living away from major human influences (e.g., major environmental disruption or exploitation of animals of interest).

**Fig. 1:**
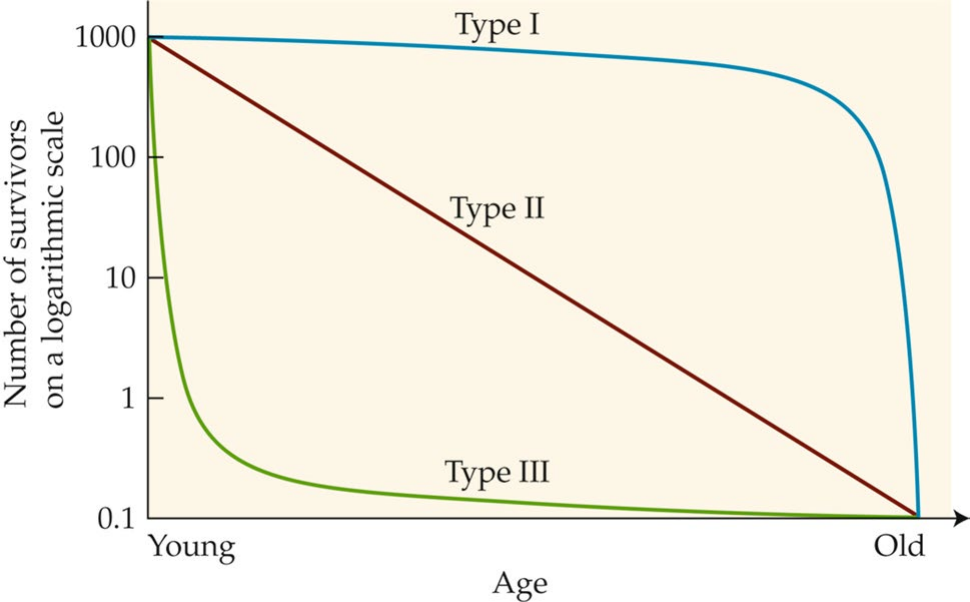
The three principal types of survivorship curve exhibited by animal populations in the wild according to Deevey (1947). Pearl and Miner (1935) presented similar antecedent curves. Each curve assumes an initial population of 1000 newly born (or hatched) individuals and shows the number of survivors as the individuals age from their time of birth or hatching to old age. Over the seven decades since its publication, Deevey’s diagram has been replicated in many textbooks and has often served as the sole or principal introduction to survivorship for generations of new biologists. Although Deevey emphasized that serious shortcomings existed in empirical support for the demography of early ages, his caveats have rarely been repeated as the diagram has been presented as a key educational tool for decades. Deevey drew the Type I curve as if no individuals died between birth and the start of high mortality in old age; here, to be more realistic while adhering to his concept, we depict slight mortality during this interval. Deevey published both axes without units; however, he noted the superiority of a logarithmic scale on the *y*-axis, and here we have added a representative logarithmic scale

The study of demography in natural populations was started seven decades ago by Edward Deevey (1947), who created the iconic diagram in Fig. 1, showing the three principal types of survivorship curve that he believed to be exhibited by animal populations in a state of nature. His diagram has been used as a major pedagogical tool ever since, appearing often in textbooks of ecology and general biology. Recognizing that most comparative physiologists—indeed, most biologists—do not undertake a professional study of demography, the Deevey diagram or some version of it is often our sole introduction to demography in the wild.

Deevey (1947), however, frankly emphasized shortcomings inherent in his diagram. Research on demography in the wild was in its infancy in 1947, and Deevey’s diagram (Fig. 1)—despite its obvious value—represented an abstraction of studies that, in his words, often had “serious deficiencies.” Deevey repeatedly highlighted one key deficiency in particular: when researchers at the time studied the survivorship of species in the wild, in almost all cases they were unable to obtain direct data on the earliest life stages. On the *x*-axis, therefore, most survivorship curves had little or no direct empirical support between age zero (0) and an age far greater than zero—a deficiency that undermined Deevey’s ability to construct his three principal curves (Fig. 1) accurately. Deevey concluded his classic essay with a clarion call: “In all cases,” he wrote, “it is the youngest ages about which we know least, and ecologists should, therefore, concentrate their efforts on this segment of the life span of animals in nature.”

Whether as a direct response to Deevey’s exhortation or as a natural next step in the advancement of demography, empirical studies of early-age survivorship in the wild started to accumulate in the 1950s and continue to accumulate. In this process, field biologists have squarely faced the often-enormous challenges of obtaining direct demographic information on the earliest life stages—with sometimes startling and unexpected insights. The empirical studies of early-age demography, however, have not previously been synthesized.

**Fig. 2:**
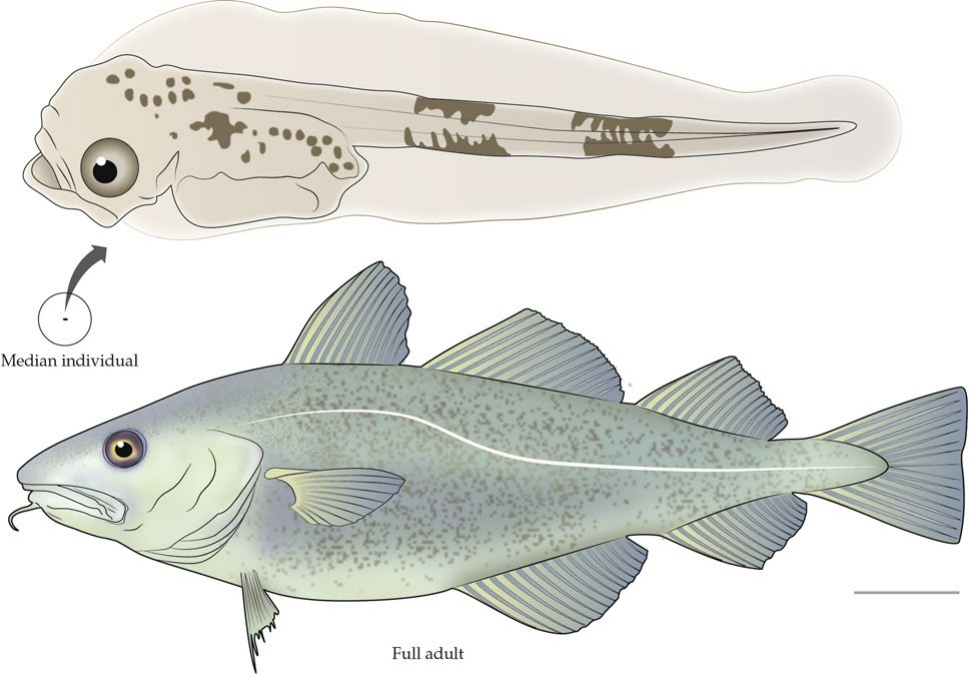
A median Atlantic cod (*Gadus morhua*) (top) compared with an adult (bottom). As described later in this paper, a “median individual” is an individual at the age when 50% of hatchlings have died. Median cod are 5–10 days old. The circled image of the median individual is at actual size relative to the adult and magnified to show detail (Auditore et al. 1994). The scale bar to the lower right of the adult represents 10 cm. The adult and actual-size median individual are scaled correctly in relation to this scale bar

An example from this literature provides a concrete illustration of the points we have made regarding the importance of demographic studies for comparative physiology, especially environmental physiology. When biologists hear the words “Atlantic cod,” they commonly envision an animal like that at the bottom of Fig. 2, about 0.5–1.0 m long: an adult. However, two empirical studies of the early-age demography of Atlantic cod (*Gadus morhua*) carried out in the decades since Deevey’s publication reveal that the median age at death of cod hatchlings in natural populations is 5–10 days. Kristiansen et al. (1997) released millions of freshly hatched, genetically marked larvae into a nearly landlocked Norwegian fjord, then followed the survival of the marked larvae. Mountain et al. (2008) monitored the demise of naturally produced larval cohorts in open water in the Western Atlantic Ocean over a 5-year period. The two studies estimated that 50% of all hatchlings died within about 5 days (Kristiansen et al. 1997) to 10 days (Mountain et al. 2008) after hatching, ages at which the fish have the size and appearance shown at the top in Fig. 2. Given that cod do not reach adulthood (attain sexual maturity) until several years old (Marteinsdottir and Begg 2002), it is clear that < 1 out of every 1 million hatchlings becomes adult. To understand cod in natural populations, physiologists might adopt the “adult perspective” and exclusively study the properties of adults. Clearly, in this case, it would be important to remember that each adult (i) passed through a stage in the first weeks of its life when its physiology was very different and (ii) survived that stage despite a crushing mortality rate. Alternatively, physiologists might adopt the “all-individuals” perspective. They might then feel compelled to study in detail the physiology of larvae aged ≤ 10 days old, given that 50% of all cod that hatch in natural populations are never-adult individuals in that category.

Here, we survey and synthesize the empirical literature on early-age survivorship in the wild that has accumulated since the publication of Deevey’s (1947) synthesis, thereby filling the gap that worried Deevey the most. We take an approach similar to Deevey’s. He examined a deliberately diverse array of animals, seeking patterns expressed throughout the animal kingdom. We also examine a deliberately diverse array of animals and synthesize within a single framework the available knowledge on these varied species.

To create such a broad synthesis, we have required an informative, universal metric that can be calculated for any population. The metric we use is the age of median individuals, defined to be individuals that live to the median age–at–death, calculated from the time of birth (or hatching). In a cohort of individuals born or hatched together, median individuals are those that live to the age at which 50% have died. Stated more generally, in any given population, median individuals live to be older than the ages-at-death of half the individuals in the population but die at an age younger than the ages-at-death of the other half.

With median individuals thus defined in terms of age, we can—as a corollary—apply the concept of “median” to physiological, biochemical, and other characteristics. Suppose, to illustrate, that the median age at death in a population is 60 days. If we could look at all the individuals ever born (or hatched) in the population when they are at the final stages of their individual lives, half would have the physiological, biochemical, and other characteristics of animals younger than 60 days; whereas, half would have the characteristics of animals older than 60 days. The physiological, biochemical, and other characteristics of 60-day olds are, therefore, in this sense, median.

Collectively, the studied populations we survey tell an impressive story of compelling importance for all biologists interested in animals in the wild. The studied populations inform us that median individuals are in general not what many biologists would assume them to be. Almost universally, whether animal populations are Type I, II, or III (Fig. 1), median individuals are young, small, and pre-reproductive throughout their lives—differing dramatically in many ways from the adults or subadults that often receive greatest attention. In Type I and II populations (Fig. 1), early mortality in the wild is far greater than Deevey imagined. Thus—even in species classically considered Type I (e.g., humans)—median individuals in a state of nature generally die at such a young age that they are never reproductive. Indeed, we conclude that Deevey’s Type I curve (Fig. 1) is misleading and must be revised: there are probably no populations in the wild that adhere even approximately to the curve he drew.

## Detailed methods

This study was planned with a single principal objective: to survey empirical knowledge of early-age demography to produce an up-to-date synthesis. In our search for research results for inclusion, we have had two primary objectives: first, base our conclusions on the most informative research available; second, maximize taxonomic diversity. To ensure that neither of these objectives would eclipse the other, we established defined taxonomic objectives from the start. We listed 17 taxonomic groups or informal subgroups: small mammals, large mammals, nonhuman primates, humans, passerine birds, nonpasserine birds, lizards, turtles, anuran amphibians, urodele amphibians, teleost fish, elasmobranch fish, insects (with the goal of including at least two orders), aquatic crustaceans, echinoderms, broadcast-spawning molluscs, and molluscs that undergo direct development. With these taxonomic groups defined, we searched the published literature to find the most rigorous empirical studies of early-age demography meeting our taxonomic targets. In the process, we strived for approximately even coverage of the 17 groups; operationally, after we had found two or three high-quality studies of a particular group, we turned our attention to other groups. In this way—within the constraints of the literature available—we achieved the best possible balance between quality of demographic research and taxonomic diversity.

For a published study of early-age demography to be included in this survey, the study had to meet the criteria listed in Table 1. The first criterion posed particular challenges because, in many published studies of early-age demography, offspring were first enumerated at ages older than birth or hatching, or at ages prior to birth or hatching (e.g., pre-ovulation egg counts). We required a rigorous attempt to enumerate offspring at birth or hatching, and all survival rates we mention are calculated from birth or hatching unless stated otherwise. To the extent possible in the modern world, we prioritized studies of populations that were relatively unexploited and living in relatively undisturbed, unpolluted environments (Table 1).

**Table 1:** Criteria that a study must meet for inclusion in this survey

∙ Empirical estimation of the number of offspring born or hatched
∙ Empirical estimation of the age at which 50% have died (“median individuals” being defined in this paper to be individuals that die at that age)
∙ Species native (i.e., not human introduced in the habitat studied)
∙ Minimal human exploitation of the studied population (especially the young age classes)
∙ Minimal human disruption or pollution of the occupied habitat

Given the current state of the world—the interconnectedness of ecosystems, the pervasiveness of human influence, and the ubiquitous practical challenges that investigators face to study animals in the wild—no studies of early-age survivorship are perfect. We have selected those studies that seem relatively best in terms of accuracy and ecological context. We have never used the results of a study to help decide whether to include the study in this survey, and no studies were omitted because of their results.

In our search of the published literature for studies meeting our criteria (Table 1), we read approximately 5 times as many published reports as we here include. This is true even though we only read papers that—based on their titles, key words, or abstracts—held promise of including useful data for meeting our objectives. We rejected 4 out of 5 papers because one or more of our criteria (Table 1) were not met—indicating that the pertinent literature is not nearly as extensive as might be hoped or surmised.

Meta-analyses of early-age demography in the wild have been published by other authors for a few taxonomic groups. Where we cite those analyses, we caution that the authors may have had different criteria than ours for including individual studies in their work.

## Illustrations of median individuals

We present four figures like Fig. 2, in which median and adult individuals of a species are depicted side by side. These figures, which have been prepared meticulously, show the body sizes of the median individuals relative to those of the adults. Body size is highly informative for physiologists. As Schmidt-Nielsen (1984) documented, it is one of the most fundamentally important properties for understanding multiple physiological attributes. Body size also strongly affects the use of physiologically protective microenvironments (Bartholomew 1964). The figures also show the morphologies of median individuals. Throughout the history of physiology, observations of animal morphology have often provided the initial impetus for innovative new physiological investigations (e.g., of sense organs, integumentary structures, and biomechanics). We view the comparative physiology of early life stages as a field in its infancy, and—as always with such fields—it is helpful to become familiar with the cast of characters.

## Survey of results

For presenting results, the 17 taxonomic groups targeted for study are grouped into larger categories (e.g., “nonhuman mammals”) for easy comparison. We take a narrative approach to presenting the results for three principal reasons. First, the challenges and methods of obtaining early-age survivorship data in the wild must be taken into account for the data to be used insightfully. There are no standardized methods. Instead, each species presents its own challenges, and almost all studies of early-age demography employ species-specific methodological innovations to be successful. With a narrative approach, we are able to present the results in ways that non-demographers are informed about the variability of methods and the sources of potential error or uncertainty. Our second reason for using a narrative approach is that high-quality demographic studies sometimes demonstrate unequivocally that the median age of death occurs within a specific, informative block of time (e.g., within a certain range of months) without estimating the median age numerically. With a narrative approach, we are able to use such results and present them rigorously. Finally, our third reason for using a narrative approach is that it allows us to include important ancillary information when it is available. Some authors, for example, point out major implications of reproductive seasonality or report direct measures of the percentages of newborns that ever have offspring. A narrative approach allows us to highlight these contributions in context.

### Nonhuman mammals

To ensure coverage of a broad range of nonhuman mammals, we made a deliberate effort (see Detailed methods) to identify high-quality studies on three categories: small mammals, large mammals, and nonhuman primates.

As is often true in studies of early-age demography, research on small mammals, such as mice and voles, must address enormous obstacles that prevent perfection (Hill 1992). Small mammals typically rear their young in highly secluded microhabitats (e.g., underground burrows or tree cavities) and take their young elsewhere or abandon them if investigators invade to inspect.

**Fig. 3:**
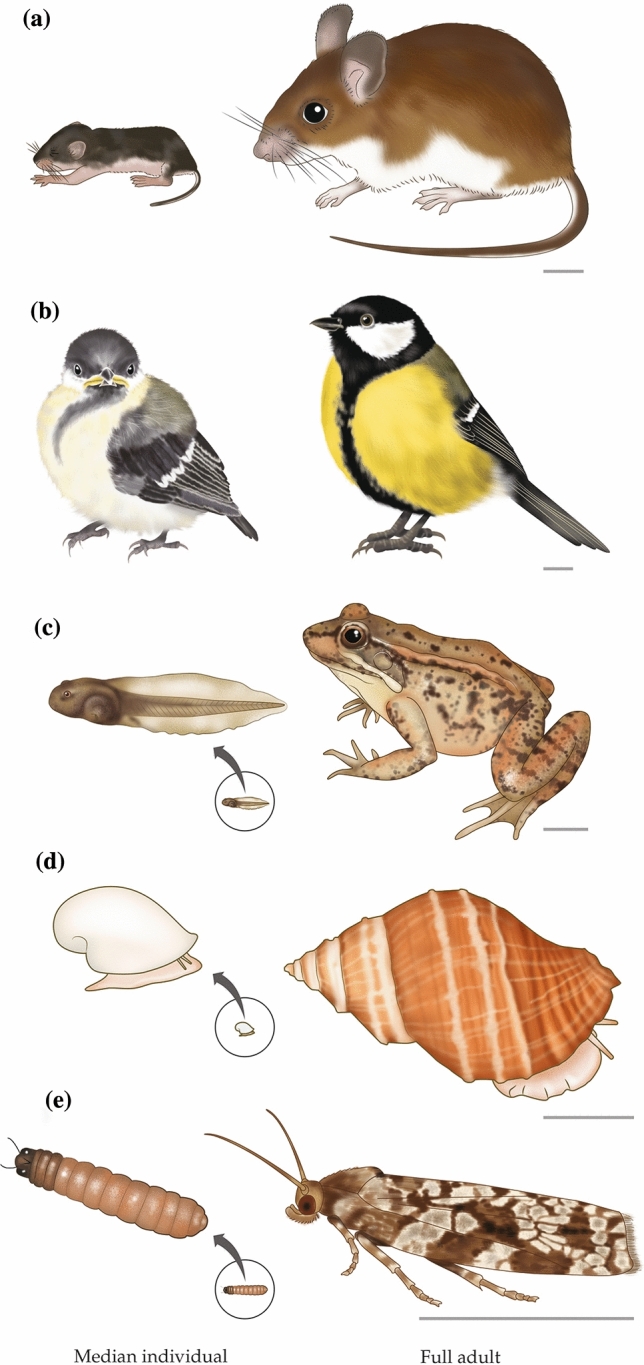
Median and adult individuals of five species. **a** white-footed mouse (*Peromyscus leucopus*). **b** Great Tit (*Parus major*; males shown). **c** northern red-legged frog (*Rana aurora*). **d** frilled dogwinkle snail (*Nucella lamellosa*). **e**, Eastern spruce budworm (*Choristoneura fumiferana*). In **a** and **b**, the median individual is shown at actual size relative to the adult. In **c**–**e**, the circled image of the median individual is at actual size relative to the adult and magnified to show detail. The scale bar to the lower right of each adult represents 1 cm

Goundie and Vessey (1986) were able to calculate a full life table for the white-footed mouse (*Peromyscus leucopus*) by capitalizing on a unique oak-hickory forest in which great numbers of wooden next boxes had been installed on a long-term basis. This native North American mouse is often the most abundant small mammal in woodlands in the central and eastern United States. In the forest studied by Goundie and Vessey, the mice nested in the nest boxes, which were designed to permit inspection in a minimally disruptive way. Investigators enumerated nestlings in the boxes and monitored young by live-trapping after weaning. Half of all newborns died by about 10 days of age, a stage of development when they were still in their nests, nursing. Thus a median white-footed mouse (Fig. 3a) was a small, relatively helpless nestling, still blind because its eyes had not opened, able to thermoregulate only when grouped with littermates in an insulating nest (Hill 1976). Eyes open at about 14 days, nestlings start making temporary excursions from the nest at about 16 days (Hill 1990), and weaning is complete at about 21 days.

John Millar (2007) conducted a meta-analysis of data on nine species of mice, voles, and lemmings, attempting to summarize early-age mortality in small mammals despite the enormous complexities of data interpretation for these elusive animals. He concluded that, on average, >50% of individuals die during the first 3 weeks of life while still nestlings. Hill (1992) synthesized data from 17 studies on total mortality between birth and 4–6 weeks of age in two *Peromyscus* species and five vole species; percent morality was 51–92%.

Among mammals of large body size, one of the most rigorous studies focused on the spotted hyena (*Crocuta crocuta*), an abundant large carnivore in many African ecosystems. Watts and Holekamp (2009) estimated gender-specific survivorship curves for a free-living population studied for >15 years using radio-collars and direct observation. Half of both the males and females born died within their first 1.2 years of life. Spotted hyenas do not attain physiological sexual maturity until 2 years old and often do not breed until older. Thus, median hyenas are never-adults that die prior to reproductive maturity. As newborn female hyenas age, 63% die before any give birth to offspring.

Ironically, the most famous life table for a large mammal is probably the one that Deevey (1947) presented for Dall sheep (*Ovis dalli*). However, as Deevey stressed and others have stressed even more (Murphy and Whitten 1976), the data that Deevey used for early ages were not dependable because the life table was based on skulls collected in the wild, and the skulls of young individuals are very perishable. Dall sheep live in rugged environments where little can be accomplished by investigators on foot directly observing the animals. Simmons et al. (1984) constructed the life table of a remote population by enumerating yearling and older individuals from the air and estimating births by examination of freshly killed females. They concluded that 51% of Dall sheep die in the first year of life, far in advance of reproductive age in either sex.

Among studies of nonhuman primates, Robinson (1988) assembled an exceptional set of data on wedge-capped capuchin monkeys (*Cebus olivaceus*) living in a protected forest in Venezuela. In our entire survey, Robinson’s study provides the only instance in which >50% of individuals lived to reproductive age—an unusual result that he observed only in females. Robinson found that 60% of female capuchin monkeys survived to 6 years of age, when reproduction began. Moreover, because of high survivorship in midlife, 50% of females survived considerably longer: A median female was a reproductively active 15- to 26-year-old adult. However, a median male was about 6 years old and far short of reproductive age (12 years).

Altmann et al. (1977), in contrast, obtained unexceptional results in their study of yellow baboons (*Papio cynocephalus*) in the Ambroseli National Park (Kenya). Half of newborns (data reported for sexes combined) died by about 10 months of age, far ahead of the ages of sexual maturity, 5–10 years.

### Humans in a state of nature

For understanding the early-age demography of human populations in a state of nature, two options exist: study of (i) ancient populations and (ii) more recent populations living apart from enhanced public health and medicine. By all indications (as we shall see), death rates of infants and children in a state of nature are so high that there are no known present-day populations that sustain themselves in the face of state-of-nature mortality schedules (Frier 2000). Details of interpretation cannot, therefore, be checked directly through study of populations alive today.

Among ancient populations, that of Roman Egypt (Egypt under the rule of the Roman Empire in CE 12-259) is of particular note because census reports from the provincial government survive, providing probably the best demographic record from the ancient world (Bagnell and Frier 1994; Scheidel 2001). The censused population, numbering 5–7 million (Scheidel 2001), consisted of farmers, laborers, soldiers, scribes, weavers, stonecutters, donkey-drivers, and other sorts of workers, plus their families (Bagnell and Frier 1994). The people were noted for raising all their children, suggesting that infanticide was not a large influence (Scheidel 2001), although infanticide cannot be excluded in any ancient population.

**Fig. 4:**
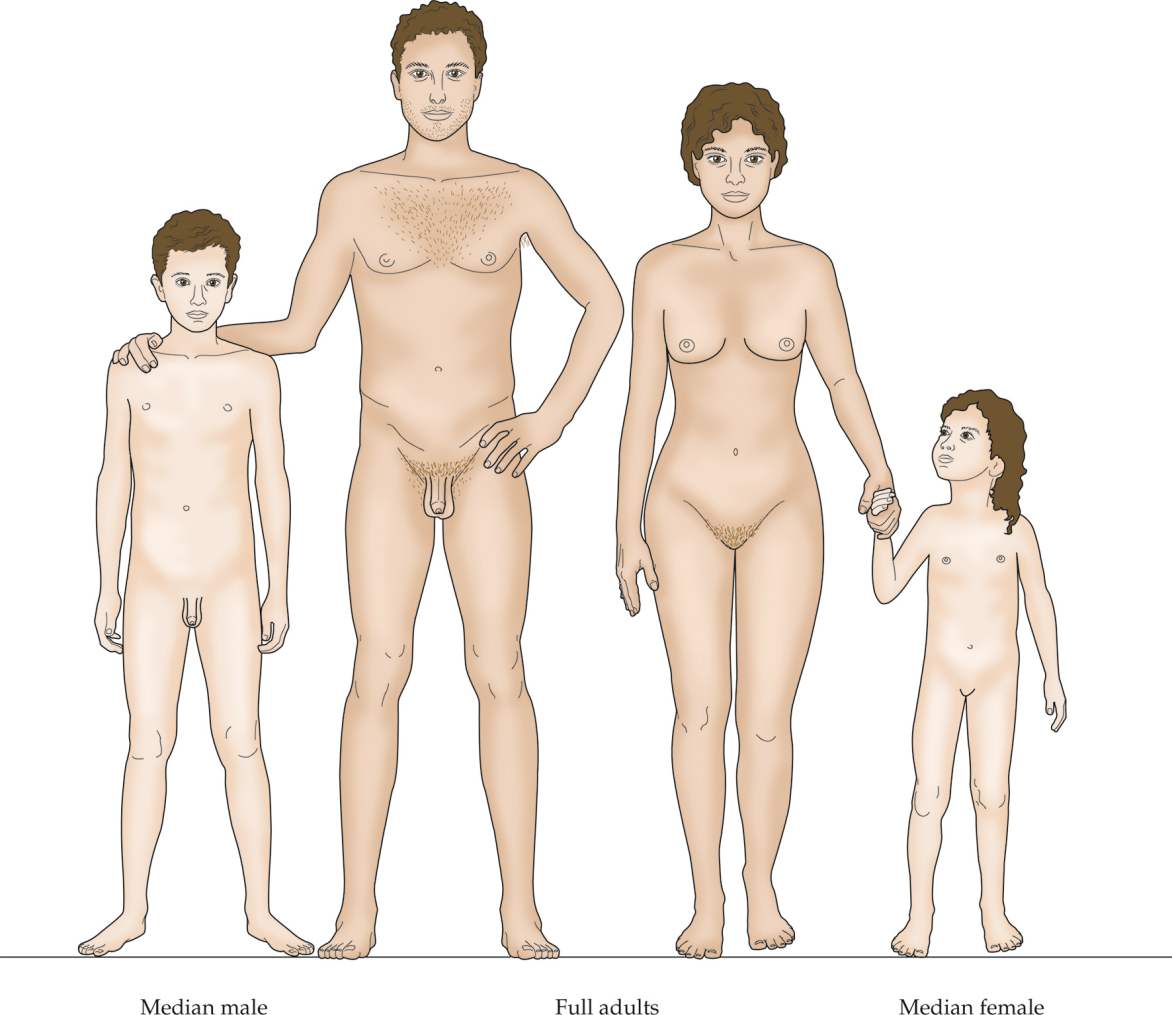
Median male and female humans shown in relation to adults in Roman Egypt (CE 12-259). Median individuals are depicted at their correct states of maturity and body sizes relative to the adults based on the life tables of Bagnall and Frier (1994)

Bagnell and Frier (1994), in their definitive, book-length analysis of demography in Roman Egypt, present full life tables for females and males. Among girls, 50% perished by approximately age 5, and among boys, 50% died by approximately age 10. Median individuals in Egypt in CE 12-259 were, thus, children as shown in Fig. 4.

For interpreting the wider research literature on human populations, it is important to recognize the necessary (although not rigid) interrelations of several demographic variables because all authors do not report their conclusions in the same way. These variables include the life expectancy at birth [*e*(0)], the early-age mortality rate, and fertility. Human demographers often employ model life tables (Coale et al. 1983) when empirical information is incomplete, and the so-called “West” family of model life tables (Coale et al. 1983) is commonly assumed to be most likely to apply to populations in a state of nature (Parkin 1992; Bagnell and Frier 1994; Frier 2000). In ancient populations, assuming “West”-type mortality schedules, an *e*(0) of 20 years was associated with 50% mortality by 5 years of age (Coale et al. 1983). An *e*(0) of 20 years, therefore, was also associated with a low proportion of female offspring reaching child-bearing age, meaning that high fertility—6-6.5 children per woman who had children—was mandatory for a population to be of stable size (rather than declining) (Hinde 2003; Livi-Bacci 2007). The three variables—*e*(0), early-age mortality rate, and mandatory fertility for population stability—tend to co-vary. For example, if *e*(0) was 25 years, 50% mortality occurred by around age 10 years (Coale et al. 1983), more girls reached child-bearing age, and the number of children each adult woman had to bear for population stability was lower. In Roman Egypt, *e*(0) was 22–25 years (Bagnall and Frier 1994).

In the Neolithic and Paleolithic periods, based on more tenuous information than available for Roman Egypt, *e*(0) is estimated to have been 17–21 years (Ferembach 1962; Livi-Bacci 2007), suggesting that the age at 50% mortality was often <5 years. Pertinent to populations between the Neolithic and Roman Egypt, Sallares (1991) analyzed a large set of cemetery data for ancient Greece (BCE 650-350) to produce a male life table. Mortality at 15 years of age was 46%. Surveying the “ancient Greco-Roman world,” Scheidel (2007) concluded (see also Parkin 1992; Frier 2000) that the most likely *e*(0) was in the lower 20s and presented a synthetic survivorship curve based on synthesis of his professional knowledge, estimating the age at 50% mortality to be 13 years. Parkin (1992) concluded that in ancient Rome, 50% died before the age of 10 years.

Besides ancient populations, another source of information on the human condition in a state of nature is study of relatively recent populations bereft of improvements in public health or medicine. Bagnall and Frier (1994) concluded that, in India and China in the early twentieth century, extremely rural populations may well have had mortality schedules closely similar to Roman Egypt. Regarding late nineteenth century Africa, John C. Caldwell (1995), an authority on African demography, concluded that *e*(0) was probably about 20 years in typical Africa societies, implying 50% mortality by 5–10 years of age. Very high rates of mortality for infants and children in a state of nature are also indicated by early field reports from sub-Saharan Africa. Beach (1990a, b), for example, analyzed reports on rural populations in the central plateau of modern-day Zimbabwe prior to 1923, when the populations were relatively stable and “remarkably healthy.” He recorded that Native Commissioners consistently reported “infant mortality” (presumably referring to infancy plus early childhood) to be about 50% of births. Taeuber (1949) summarized field reports from Tanganyika (now Tanzania) in 1910–1930. The Medical Department for Tanganyika Territory, for example, reported that in eight districts near Tabora in 1922, 45–66% of newborns died in childhood. For 2300 women in Ulanga, > 50% of children died before puberty.

The two sorts of fragmentary data emphasized here—data from ancient populations and from more-recent populations before modern demographic improvements—converge to tell a remarkably consistent story. Namely, children in human populations in a state of nature probably suffered 50% death by roughly the age of 10 years. To be somewhat more conservative, we might say 50% died before reaching puberty, as Scheidel (2009) has eloquently emphasized in regards ancient populations. In a state of nature, a median person was a child or prepubescent teenager (Fig. 4), and < 50% of individuals ever attained reproductive maturity.

### Birds

The norm in passerine birds is for >50% of nestlings to survive from hatching to the time they fledge from the nest, as Ricklefs (1969) established in his early, extensive review of nestling demography. For estimation of the age of 50% mortality, therefore, the focus shifts to the period following fledging, a particularly challenging part of the life cycle for quantitative study of mortality because fledglings scatter in unpredictable ways.

The Great Tit (*Parus major*) is one of the most intensely studied birds in the world. Native to the woodlands of Europe and parts of Asia, it is typically nonmigratory. To estimate the age of 50% mortality in Great Tits in the wild, we have used two studies in Sweden that together provide data on nestling (Dufva and Allander 1996) and fledgling (Dhondt 1979) survivorship. Based on these studies, 50% of all Great Tits that hatch are dead by about 6–7 weeks of age. That is, median individuals are fledglings about 3–4 weeks after leaving the nest. They are nearly the same size as adults, as Fig. 3b shows, but differ from adults in bill structure, color brightness, and color patterns—in addition to exhibiting behavioral and physiological differences such as being reproductively immature.

Considering a median, 6- to 7-week-old Great Tit, how much older must it become to reproduce? In natural populations, the answer depends strongly on reproductive seasonality (an effect that we illustrate here but that has broad implications). Because of the controls on reproductive seasonality, Great Tits typically breed in the spring (April–May). Thus, an individual that fledges in one year is unlikely to breed (and thereby achieve full adulthood) until the following spring. This seasonal programming greatly increases the time that must pass for a median individual to reach adulthood. It, thus, also greatly increases pre-adult mortality because death is an ever-present danger as time passes. Kluijver (1951) concluded that only about 13% of hatchling Great Tits ever breed, with overwintering mortality adding substantially to the total mortality.

Recently, two meta-analyses of post-fledging mortality in the wild in passerine birds have been published. Both pull together data on dozens of species. Cox et al. (2014) report that, on average, 40% of passerine fledglings die in the first 3 weeks following fledging. Naef-Daenzer and Grüebler (2016), who include altricial nonpasserines as well as passerines, conclude that about 25% of individuals that fledge die in the first 4 weeks afterward. Combined with mortality between hatching and fledging (Ricklefs 1969) and mortality associated with seasonal programming, these levels of early fledgling mortality virtually guarantee that total pre-adult mortality considerably exceeds 50%.

Among nonpasserine birds, demography in the nest is extremely variable according to Ricklefs’ (1969) review. Suffice it to say that Ricklefs’ nestling mortality data alone provide useful insights. For example, in half of the 12 studies of larids (gulls and terns) tabulated by Ricklefs, > 50% of hatchlings died before fledging. It, thus, seems common among larids that median individuals are nestlings.

### Amphibians, lizards, and turtles

Among amphibians, >50% of hatchlings typically die prior to metamorphosis, meaning that median individuals are tadpoles. Calef (1973), for example, studied survivorship in northern red-legged frogs (*Rana aurora*) occupying a small, pristine lake in the mountains of British Columbia. Each year, adult frogs enter the lake to breed soon after ice disappears, and eggs are soon produced. Among the approximately 300,000 hatchlings that result, only 50% are still alive at about 3 weeks of age, when the hatched individuals are young tadpoles, as shown in Fig. 3c. Metamorphosis does not start for another ca. 8 weeks, and only about 1 hatchling out of 20 undergoes metamorphosis. Unlike Calef, Herreid and Kinney (1966) marked animals with dye, a method that conceivably could have affected results. That said, in their study of woodfrogs (*Lithobates sylvaticus*) in four Alaskan ponds, they found the stage of development at 50% survival to be similar to that reported by Calef (Fig. 3c). Among the numerous studies published on salamanders (urodeles) in the genus *Ambystoma*, many do not clearly distinguish pre- and posthatching mortality. A study that seems particularly clear in distinguishing them is that by Peterson et al. (1991) on ringed salamanders (*Ambystoma annulatum*). In each of two small ponds, survivorship of hatchlings fell to <50% within the first month after hatching, far in advance of metamorphosis.

Tinkle (1967) conducted a thorough study of side-blotched lizards (*Uta stansburiana*) in a Texas desert. Although he marked the animals, he took steps to document that the marking did not affect results significantly. He found that 50% of both males and females died in the first 4 weeks following hatching, having attained ≤20% of adult weight. Although sexual maturation occurred in “a few months,” young did not reproduce until the year after they hatched, at ca. 9 months of age, because of seasonal programming. Thus, in comparison with reproducing adults, median lizards were quite young and small.

Although terrestrial turtles present exceptional challenges for quantification of early-age survivorship, data providing valuable insight are available. The studied species live in ponds or wetlands in adulthood, and they have been investigated using fences erected around the bodies of water, turtles being enumerated as they navigate the fences. The fences have a key limitation: Hatchling turtles—which hatch on land—are first enumerated as they arrive at the fences, when they have already suffered some unmeasured posthatching mortality. Insight has been gained, nonetheless, by looking at survivorship following first enumeration by a fence. Based on large sample sizes, Iverson (1991) and Frazer et al. (1991) estimated survivorship over the year following first fence detection in two species of mud turtles (*Kinosternon* spp.): 19% survival in *K. flavescens* in Nebraska, 16% in *K. subrubrum* in South Carolina. Survivorship from hatching would have been even lower. Recognizing that the turtles do not reach sexual maturity until 11 and 4 years old, respectively, it is clear that in both species, 50% of hatchlings died at ages far in advance of adulthood.

### Elasmobranch fish (sharks, skates, and rays)

We have already mentioned cod and will return to the teleost fish shortly when we summarize mortality in broadcast spawners. Here, we discuss the elasmobranch fish, which are not broadcast spawners and differ radically from the teleosts in their early lives. Whereas teleosts exhibit indirect development (so-called because they hatch as larvae), elasmobranchs undergo direct development, meaning that—in body form—their newly hatched or newly born offspring are sizable “small adults.” Some elasmobranch species (classed as oviparous) lay eggs, provisioned with abundant yolk, in which extensive prehatching development occurs. Other elasmobranchs (viviparous) retain young for extended periods of development within the mother’s reproductive tract prior to birth. In either case, when the young hatch or are born—although immature—they are far larger than teleost hatchlings and have an adult-like appearance.

Few studies have been conducted on the early-age demography of elasmobranchs. Nonetheless, careful studies have been appearing, including studies on two viviparous species, lemon sharks (*Negaprion brevirostris*) and tiger sharks (*Galeocerdo cuvier*). Gruber et al. (2001) estimated the survivorship of young lemon sharks in an unexploited population. Half died within the first 2 years following birth, probably within the first year. Median individuals were, thus, 1–2 years old—meaning they were sexually immature and <1 m long—whereas adults are ≥12–13 years old and reach 2–3 m. Similarly, Driggers et al. (2008) documented that in tiger sharks, the age at which newborns exhibit 50% survival is 1–2 years. Median individuals are thus far younger than reproductively mature adults (≥10 years of age).

### Direct-developing aquatic snails

Like fish, the aquatic molluscs include some species that undergo direct development even though most species (discussed later) exhibit indirect development. To gain insight into the early-age demography of direct-developing molluscs, Spight quantified early mortality in multiple species of ocean-dwelling, direct-developing carnivorous snails, principally drill snails in the genus *Nucella* (*Thais*). Adult female *Nucella* produce egg capsules (each containing multiple eggs) that they attach to rocks. At hatching, tiny snails crawl out on the rock surface, where they feed throughout their postemergent development. In the frilled dogwinkle (*N. lamellosa*), we calculate from Spight (1975, 1976) that 50% of hatchlings die in 14–16 days. Thus, as seen in Fig. 3d, median individuals—although having the appearance of snails—are far younger and smaller than reproductive adults, which are ≥4 years old. Other *Nucella* species are similar.

### Insects

The spruce budworm (*Choristoneura fumiferana*), a species of moth native to North America, lives in spruce and fir forests, sometimes causing devastating damage because its larvae (caterpillars) feed on buds, foliage, and flowers as they pass through their four developmental stages (instars I–IV) prior to pupation. An advantage of the species for demographic research is that each year’s production of eggs is almost synchronous; a disadvantage is the need to sample throughout the height of 20-m-high forests. Morris and Miller (1954) constructed a life table for a budworm population living in a stand of fir trees in New Brunswick, Canada—a stand where the insect population was not overly dense and not being sprayed with insecticides. After individuals hatched from eggs, 50% died while in larval instar I or II, when they were about 2 mm long. Thus, a median individual was a small, relatively young caterpillar (Fig. 3e). Only about 1 out of 200 hatchlings reached adulthood; 199 were never-adults.

Another instructive study, on a different insect order, was Danthanarayana’s (1969) research on *Sitona regensteinensis*, a native weevil (beetle) in England that lives principally on one plant, broom. Because the adult weevils live aboveground but the larvae feed on underground plant parts, study of the life cycle demanded sampling in two habitats. Only 14–15% of hatchlings survived the early part of the first larval instar. Thus, a median individual was a young larva living in an entirely different habitat from adults.

Cornell and Hawkins (1995) and Price (1997) compiled meta-analyses of survivorship in large numbers of herbivorous insect species (approximately 154 and 29 species, respectively). Some species were studied in agricultural fields, limiting applicability to our survey of survivorship in the wild even though the fields were not being sprayed with insecticides. Moreover, Cornell and Hawkins emphasized that the species in their analysis were not a random sample of insects but instead tended to be ones with highly apparent, persistent populations. In the majority of species in these meta-analyses, ≥ 50% of hatchlings died at very early ages, and a median individual was an early-stage larva, similar to the spruce budworm (Fig. 3e) and *Sitona* weevil. However, in significant numbers of other species, a median individual was a late-stage larva or pupa. In no cases was it clear in a wild population that a median individual had attained adulthood.

### Broadcast-spawning aquatic animals

We address these animals last because they are the ones that were most accurately understood (qualitatively) prior to the modern study of early-age demography. Already by 1935, Pearl and Miner offered a qualitative hypothesis, postulating that broadcast-spawning aquatic animals in the wild experience intense early-age mortality and follow a survivorship curve shaped like Deevey’s Type III (Fig. 1). All biologists learn this concept in their basic education. It is a qualitative concept, however, and notably—because of lack of data—neither Pearl and Miner (1935) nor Deevey (1947) attempted to quantify the *x*-axis for wild populations.

The advance of early-age demography since Deevey’s publication has provided a number of examples in which quantitative estimates of the early-age mortality rate are reasonably constrained. This advance has, thus, greatly refined understanding of these animals.

Atlantic cod (*Gadus morhua*) are one of the mostly intensively studied teleost fish. In wild populations, as we have seen, two careful measures on opposite sides of the Atlantic Ocean indicate that 50% of newly hatched individuals die within 5–10 days (Kristiansen et al. 1997; Mountain et al. 2008), meaning that median individuals are tiny, early-stage larvae (Fig. 2). Houde (1997) conducted a meta-analysis of empirical data on early-age survivorship in wild populations of five species of marine teleost fish besides cod. He recognized a series of developmental stages and calculated stage-specific estimates of survivorship. In all five species, the first stage following hatching lasted 3–10 days, and > 50% of hatchlings died during that stage. Thus, median individuals in all five species are < 10 days old. Houde (2002) later produced a conceptual plot of the typical lifetime survivorship curve in marine teleost fish. This plot projects that, as observed in cod, < 1 out of every 1 million hatchlings reaches adulthood, emphasizing that adults are not typical individuals in populations of these highly fecund animals. Freshwater teleosts exhibit moderately lower early death rates than marine teleosts, but not low enough to substantially alter this picture (Houde 2002).

**Fig. 5:**
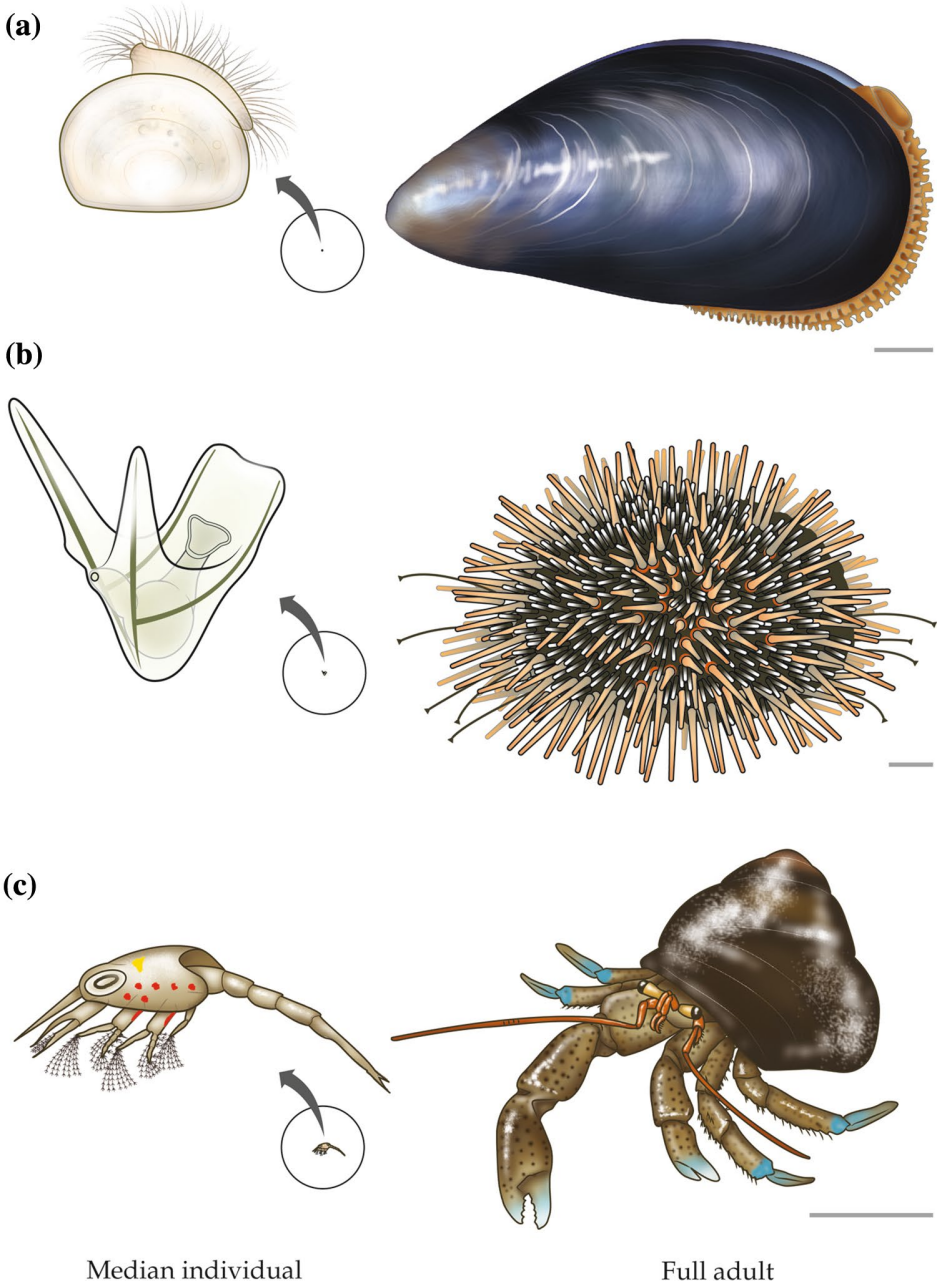
Median and adult individuals of three marine, broadcast-spawning invertebrates. **a** blue mussel (*Mytilus edulis*). **b** kina sea urchin (*Evechinus chloroticus*). **c** blueband hermit crab (*Pagurus samuelis*). In each case, the circled image of the median individual is at actual size relative to the adult and magnified to show detail. The scale bar to the lower right of each adult represents 1 cm

Regarding broadcast-spawning invertebrates, Jørgensen (1981) deserves credit for being one of the first to meet the challenge of quantifying early-age survivorship in the wild. He took advantage of a synchronous spawning event in blue mussels (*Mytilus edulis*) in a relatively isolated body of water (Isefjord, Denmark) to quantify the early-age mortality rate. By his estimate, 50% of hatchlings died in 5 days. Median individuals were, thus, extremely tiny, early-stage veliger larvae as seen in Fig. 5a. To quantify early-age survivorship in another broadcast-spawning invertebrate, the kina sea urchin (*Evechinus chloroticus*), Lamare and Barker (1999), in New Zealand, also capitalized on a synchronous spawning event in an isolated locale. Based on their consensus measure of mortality rate, 50% of the hatchlings died in 4 days. A median urchin was, thus, a tiny, early-stage pluteus larva (Fig. 5b).

The study of early-age mortality in the small offspring of invertebrate broadcast spawners in natural waters is, of course, a challenge subject to considerable potential error. Still today, therefore, investigators seek new, more accurate approaches. White et al. (2014) are among the teams innovating new methods. They argue that in general, their methods yield mortality rates that are lower and more accurate than other methods. Working in the inshore waters of the mid-California seacoast, White et al (2014) sampled with plankton nets every other day for two months to estimate larval mortality rates in several crustacean taxa, including *Pagurus*, a genus of hermit crabs. One of the principal species was the blueband hermit crab (*P. samuelis*), and to visualize their results we apply their *Pagurus* mortality rates to that species. Keep in mind that White et al. deem mortality rates estimated by their method to be relatively low. They estimate 50% mortality by 11 days after hatching. A median blueband hermit crab is, thus, an early (second-stage) zoea larva of the size and body form (MacMillan 1971) shown in Fig. 5c.

From the cases we discuss here, a pattern emerges in broadcast-spawning aquatic animals, both vertebrate and invertebrate: Median individuals are tiny larvae, < 1–2 weeks old, of very different body form from adults (Figs. 2, 5). Rumrill (1990) and Gosselin and Qian (1997) present meta-analyses that quantify results in a variety of ways.

## Overview and conclusions

Of all the properties of individual animals of interest to comparative physiologists, age and stage of development are among the most consequential (Hill 1976, 2017; Fuiman and Batty 1997; Matoba et al. 2000; Mortola 2001; Fuiman and Werner 2002; Lestyk et al. 2009). For environmental physiologists and others interested in natural populations of a species, the survivorship curve provides invaluable information because it reveals the relative abundances of ages and stages of development. Demography in the wild is, thus, important for the study of comparative and environmental physiology.

We have examined early-age survivorship data for populations of a wide diversity of animal species living in the wild in relatively untrammeled environments, always expressing survivorship relative to the numbers of individuals born or hatched. As our reviews of demographic studies have repeatedly revealed, the challenges of studying early-age survivorship are often severe. Moreover, these challenges vary dramatically from taxon to taxon; for example, the challenges of monitoring life and death in newly born mice in a woodlot are almost completely different from the challenges of studying newly hatched fish in the open ocean, and both are almost completely different from the challenges of studying newly hatched insects in a 20-m-high fir forest. Successful studies, therefore, have typically depended on species-specific methodological innovations. Important generalizations emerge nonetheless, we have found. In virtually all species, 50% of individuals die before reaching even the early stages of adulthood. In fact 50% usually die far in advance of the onset of adulthood. These conclusions are true for essentially all life histories: whether we consider humans or mice, insects or birds, aquatic animals or terrestrial ones, or species undergoing direct or indirect development (e.g., elasmobranchs versus teleosts).

This survey is in many ways a logical sequel to Deevey’s (1947) seminal analysis that started the modern study of demography in “natural populations” (his term). Deevey examined populations ranging from barnacles to rotifers to birds to mountain sheep, seeking patterns expressed throughout the animal kingdom. He confronted severe deficiencies in the empirical data available on early ages, however, and in this sequel, we address the deficiencies by surveying the many hard-won advances in early-age demography made in intervening years. Like Deevey, we have examined a deliberately diverse array of animals, and we have sought patterns expressed throughout the animal kingdom.

Most individuals in most populations, we have found, lead entirely pre-adult lives. They are never-adults. In teleost fish, it is common for each 1 million offspring hatched to include roughly 999,999 never-adults and 1 adult (Houde 2002). In Great Tits, among every 100 individuals hatched, 87 are to become never-adults and 13, adults (Kluijver 1951). In humans when they lived in a state of nature, out of every 100 newborns, about 53 were destined to be never-adults, whereas about 47 reached adulthood (Bagnall and Frier 1994).

These attention-getting numbers raise the question of whether comparative physiologists should feel compelled to direct increased attention to the physiology of young stages. Obviously, however, the numbers themselves do not answer the question. Adults can easily be argued to deserve exceptional attention because, compared with individuals in early life stages, they have more ecological influence per individual (e.g., because of reproduction). Moreover, physiologists and other biologists set priorities on the basis of multiple considerations, not just relative numbers of individuals.

What, nonetheless, are the implications of the demographic data, which—in harmony with physiologists’ general commitment to quantification—quantify the numbers of individuals in the sequential stages of postnatal development? As outlined in the Introduction, the implications depend on which of two perspectives physiologists adopt: the “adult” perspective or the “all-individuals” perspective.

When the adult perspective is adopted in research on a species, the physiology of adults is investigated, and the properties of the early life stages are placed in the background. If the early life stages are considered at all, they are viewed as stages that each adult passed through as it developed into an adult. Demographic data are relevant because they potentially provide insight into the rigors that adults faced as they passed through the preadult stages of their lives. Advances in research in the past 15 years point in two ways to mechanisms by which those rigors in early life might have affected the physiological characteristics of the adults studied. These advances relate to natural selection and epigenesis. Despite the traditional view that early mass mortality in broadcast-spawning aquatic animals acts indiscriminately, evidence has been accumulating that in fact, during such die-offs, natural selection can occur in ways that affect the characteristics of later life stages (Gagliano et al. 2007; Perez and Munch 2010). The revolution in epigenetics is also pertinent. For example, it is now known that nestling lab rats, as a result of the properties of their nestling physiological systems, respond to maternal care behaviors in ways that epigenetically alter the function of their adult hypothalamic–pituitary–adrenal axis (Weaver et al. 2004). Similarly, when zebrafish (*Danio rerio*) are exposed as larvae to a number of environmental toxicants at concentrations so low that no immediate effects can be detected, the fish are subject to multiple, epigenetically mediated alterations in their adult physiology and behavior (Aluru 2017; Aluru et al. 2017).

The alternative to the adult perspective is adopting an all-individuals perspective in which all life stages are considered to be of direct interest. Schmidt-Nielsen’s (1972) aphorism—that physiology is the study of “how animals work”—is then pertinent. Individuals in all life stages are animals. The ways that all life stages “work” should, therefore, be of interest to physiologists. In this perspective, we might even see the great numbers of never-adults as a direct justification for studying them.

Certainly, regardless of perspective, it seems shortsighted to omit study of postnatal ontogeny from definitions of comparative physiology. In one of the most recent essays on the nature of modern comparative physiology, Mykles et al. (2010) stress that comparative physiology pursues integration on a “grand” scale. However, in their otherwise laudable essay, they itemize the types of integration as “vertical integration of physiological processes across organizational levels within organisms, horizontal integration of physiological processes across organisms within ecosystems, and temporal integration of physiological processes during evolutionary change.” Integration across the sequential stages of each individual’s postnatal life is just as important and belongs on any short-list of comparative physiology’s goals. Indeed, Lestyk et al. (2009) demonstrate elegantly how a well-conceived, comparative study of physiological and morphological ontogeny can contribute directly to integration in the other dimensions.

Because of its intrinsic interest to us, the human condition in a state of nature deserves brief note. No human populations living in this state are extant in the modern world. Nonetheless, as biologists, anthropologists, and others reason about the evolution of human physiological traits, it is important to remember that for much of human evolution, state-of-nature demography prevailed. Suppose we assume that *Homo sapiens* has been evolving for 200,000 years (Galway-Witham and Stringer 2018) with a generation time of 22 years—thus about 9090 generations. All the data available on human demography in a state of nature point to a single inescapable conclusion: for about 9045 of those generations, the lives of ≥ 50% of individuals ended when they were infants, children, or prepubertal teenagers. Yet this salient point is rarely, if ever, considered in theorizing about human evolution. Just in considering neurophysiological evolution, it is important to recognize that never-adult children and teenagers in a population are not merely passive actors who function in ways driven entirely by the adults in their midst. They play active roles in their own individual fates and sometimes in the fates of their societies, as when pastoralist societies give children and prepubertal teenagers full responsibility for their livestock herds, a responsibility of truly life-and-death importance for their families (Sieff 1997).

Our survey demonstrates unequivocally that the Type I survivorship curve in Deevey’s (1947) iconic family of curves (Fig. 1) is incorrect and must be revised. The necessity of revision is a serious matter for all comparative biologists, considering how commonly the Deevey diagram features in pedagogy intended to provide biologists with a useful introduction to demography.

As drawn, Deevey’s Type I curve (Fig. 1) indicates that there is a significant category of animal species in which offspring in natural populations undergo only minor mortality prior to adulthood. Stated more rigorously, Deevey’s Type I curve, as his drew it, indicates that in natural populations of such animals, the early life stages survive so well that attrition in early life is as low as that in mid-life.

Based on our survey, this situation never (or almost never) occurs in natural populations. That is, the Type 1 survivorship curve described by Deevey has very few or no actual analogs in the real, natural world. In drawing his Type I curve (Fig. 1), Deevey was highly influenced by a similar curve in Pearl and Miner (1935) that described laboratory populations, not natural ones. Thus, although Deevey’s goal was to describe “natural populations,” he drew his Type I curve (Deevey 1947) in a way that applies only to animals in human-modified protective environments, such as fruit flies in jars, farm animals on farms, dogs lounging about in living rooms, or human populations with modern public health (Scheidel 2007).

**Fig. 6:**
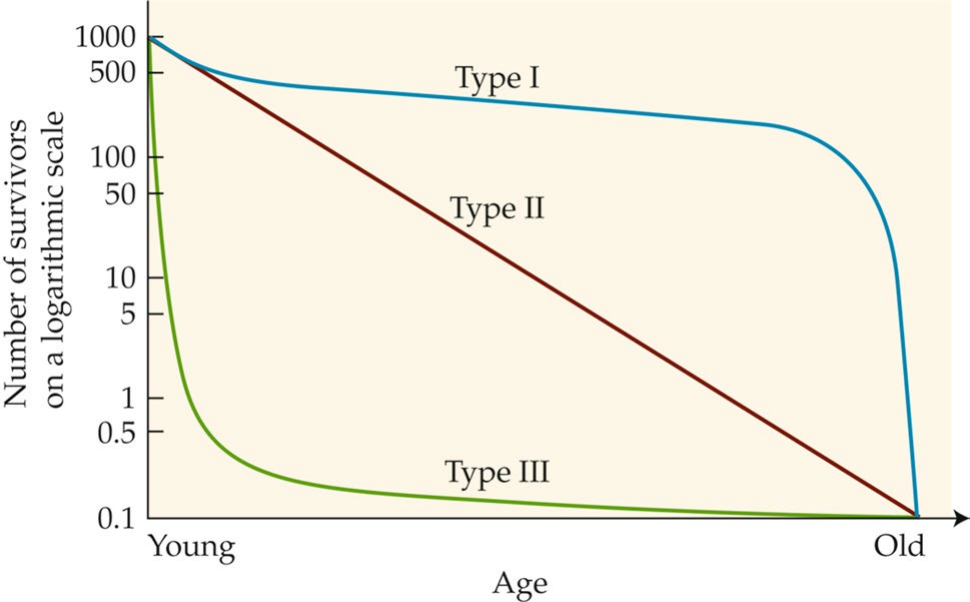
The survivorship curves presented by Deevey (1947) for animals in the wild, with the Type I curve revised to show 50–60% mortality in early life. Compare with Fig. 1. The revision of the Type I curve reflects modern evidence on early-age survivorship in natural populations. Along the *y*-axis, intermediate values are added to the logarithmic scaling (compare Fig. 1) to aid interpretation. As were Deevey’s original curves, the curves shown are conceptual and generalized, not intended to describe any particular species

For Deevey, the three survivorship curves (Fig. 1) were conceptual; he did not calculate them mathematically from survivorship data on natural populations. In the spirit of presenting a revised conceptual curve, we would re-draw the Type I curve as in Fig. 6. This revised curve reflects the fact that in species likely to qualify as Type I in the wild (e.g., humans and other large mammals in a state of nature), survivorship typically falls to ≤ 50% relatively early in the interval between birth and old age and then levels prior to a steep decline later in life. The human case in a state of nature provides a quantitative example. In Roman Egypt, if the data in the life tables constructed by Bagnall and Frier (1994) are plotted, they resemble the Type I curve in Fig. 6. Similarly, Scheidel (2007) presents an estimated curve for humans in a state of nature (his Fig. 3.1) that, for much of the age range, resembles the Type I curve in Fig. 6.

The revised conception of the three principal survivorship curves in Fig. 6 emphasizes the principal finding of our survey. Large proportions of never-adults are present in all animal populations in the wild.
